# The Use of Next-Generation Sequencing to Assist in the Diagnosis of Atypical Vasculitis

**DOI:** 10.7759/cureus.73247

**Published:** 2024-11-07

**Authors:** David A Chetrit, Thanda Aung, Quen J Cheng, Jennifer King

**Affiliations:** 1 Division of Rheumatology, Carolina Health Specialists, Myrtle Beach, USA; 2 Department of Medicine, Division of Rheumatology, University of California Los Angeles David Geffen School of Medicine, Los Angeles, USA; 3 Department of Medicine, Division of Infectious Diseases, University of California Los Angeles David Geffen School of Medicine, Los Angeles, USA

**Keywords:** cerebral infarction, infection, next-generation sequencing (ngs), prevotella melaninogenica, vasculitis

## Abstract

Vasculitis can be challenging to diagnose, especially when vessels of multiple sizes are affected and manifestations do not classically fit into defined rheumatic disease entities. We present the case of a 58-year-old Caucasian woman who presented with headache and altered mental status, with subsequent left-sided hemiparesis and hemispatial neglect eight days after a dental procedure. She was found to have extensive multi-focal ischemic infarctions secondary to vasculitis affecting multiple intracranial blood vessels. Subsequent imaging showed increasing involvement of intra-abdominal blood vessels. There was no evidence of endocarditis. Serologies for lupus and autoimmune rheumatologic diseases were unremarkable. Our suspicion was high for an infectious trigger of vasculitis. However, extensive conventional diagnostic testing, including multiple blood cultures and brain biopsy, did not reveal an underlying infectious etiology. The use of next-generation sequencing (NGS) of cell-free DNA from blood revealed the infectious pathogen *Prevotella melaninogenica*. Combining targeted anti-microbials with systemic steroids, plasmapheresis, and immune suppressant therapy, this case had a favorable outcome. The use of NGS can be useful in the diagnosis of atypical vasculitis.

## Introduction

Vasculitides are a group of disorders characterized by inflammation of blood vessel walls, which can lead to vessel occlusion, ischemia, and end-organ damage [1]. The etiologies are diverse, ranging from autoimmune processes to infections [2]. Accurately diagnosing the underlying cause is crucial, as treatment with immunosuppressive therapy may exacerbate infections masquerading as vasculitis [2].

Autoimmune vasculitides result from dysregulation of the immune system with the production of autoantibodies or immune complexes that attack vessel walls [3]. Infectious vasculitides can occur from direct vessel invasion by pathogens, such as bacteria, viruses, fungi, or parasites, or from immune complex deposition on vessel walls during systemic infection [4].

We present a case of a woman with rapid neurological deterioration and multi-vessel infarcts following a dental procedure, with systemic involvement indicating large- and medium-vessel vasculitis. Despite an extensive workup, the underlying infectious etiology was only identified using next-generation sequencing (NGS) of cell-free circulating DNA [5]. This case highlights the pivotal role of recent advances in molecular diagnostics in solving atypical clinical presentations.

## Case presentation

A 58-year-old Caucasian woman presented with altered mental status. Three weeks prior to admission, she underwent a gum grafting dental procedure. Eight days postoperatively, she developed severe headaches, neck pain, nausea, and photophobia. She became disoriented, prompting her presentation to the emergency department for further evaluation. On admission, the patient was hypotensive and afebrile. She was oriented to person only, with short-term recall difficulty. Initial labs showed a normal CBC with differential, complete metabolic panel, and an elevated ESR of 52 mm/hour. An MRI (magnetic resonance imaging) of the brain showed acute multifocal infarcts and leptomeningeal enhancement, concerning for a cardioembolic phenomenon and/or meningitis. Vancomycin, ampicillin, ceftriaxone, and acyclovir were started. A lumbar puncture was obtained (Table 1). The MR angiogram showed multifocal stenoses involving the M2 and A2 segments and the basilar artery and indicated an aneurysm at the terminus of the right ICA (internal carotid artery). Transthoracic and transesophageal echocardiograms showed no vegetation. Antibiotics were later discontinued as CSF (cerebrospinal fluid) cultures were negative, and her examinations were deemed inconsistent with an infectious disease. Acyclovir was discontinued after VZV (varicella-zoster virus) and HSV (herpes simplex virus) PCR returned negative.

**Table 1:** Results of laboratory tests SM Ab: Smith antibody; RNP Ab: ribonucleoprotein antibody; SSA: Sjögren's-syndrome-related antigen A; SSB: Sjögren's syndrome type B; C-ANCA: cytoplasmic antineutrophil cytoplasmic antibody; P-ANCA: perinuclear antineutrophil cytoplasmic antibody; RBC: red blood cell; WBC: white blood cell; CSF: cerebrospinal fluid; IgG: immunoglobulin G

Variable	Value	Reference Range
White blood cells	8.54	4.16 9.95x10^3^/uL
Hemoglobin	12.1	11.6-15.2 g/dL
Platelet count	365	143-398x10^3^/uL
Sodium	141	135-146 mmol/L
Potassium	3.6	3.6-5.3 mmol/L
Chloride	106	96-106 mmol/L
Total CO_2_	23	20-30 mmol/L
Anion gap	12	8-19 mmol/L
Total protein	7.5	6.1-8.2 g/dL
Albumin	4	3.9-5.0 g/dL
Total bilirubin	0.4	0.1-1.2 mg/dL
Direct bilirubin	< 0.2	≤ 0.3 mg/dL
Alkaline phosphatase	66	37-113 U/L
Aspartate aminotransferase	16	13-47 U/L
Alanine aminotransferase	18	8-64 U/L
Erythrocyte sedimentation rate	52	≤ 25 mm/hour
C-reactive peptide	0.7	< 0.8 mg/dL
C3	151	76-165 mg/dL
C4	34	14-46 mg/dL
Antinuclear Ab	< 1:40	< 1:40 titer
dsDNA Ab EIA	≤ 200	≤ 200 IU/mL
SM Ab	< 20	< 20 U
RNP Ab	< 20	< 20 U
SSA antibody	< 20	< 20 U
SSB antibody	< 20	< 20 U
Rheumatoid factor	< 10	< 25 IU/mL
MI-2 autoantibodies	Not Detected	Not Detected
Cyclic citrulline Ab IgG	5	≤ 19 U
Thyroid peroxidase antibody	116	≤ 20 IU/mL
Beta-2-glycoprotein IgA	2	0-20 SAU
Beta-2-glycoprotein IgG	0	0-20 SGU
Beta-2-glycoprotein IgM	21	0-20 SMU
Cardiolipin IgA	< 12	< 12 APL
Cardiolipin IgG	< 15	< 15 GPL
Cardiolipin IgM	< 12.5	< 12.5 MPL
C-ANCA	< 1:20	< 1:20 titer
Myeloperoxidase Ab	< 20.0	< 20.0 CU
P-ANCA	< 1:20	< 1:20 titer
Proteinase-3 Ab	< 20.0	< 20.0 CU
RBC count, CSF	2,000	0-10/cmm
WBC count, CSF	10	0-5/cmm
Segmented neutrophils	10	< 25%
Lymphocytes	82	40-80%
Monocytes	7	15-45%
Basophils	1	0-0%
Glucose, CSF	57	43-73 mg/dL
Protein, CSF	50	15-45 mg/dL
Oligoclonal bands, CSF	0	0-1 bands
IgG index	0.6	0.0-0.6

**Figure 1:**
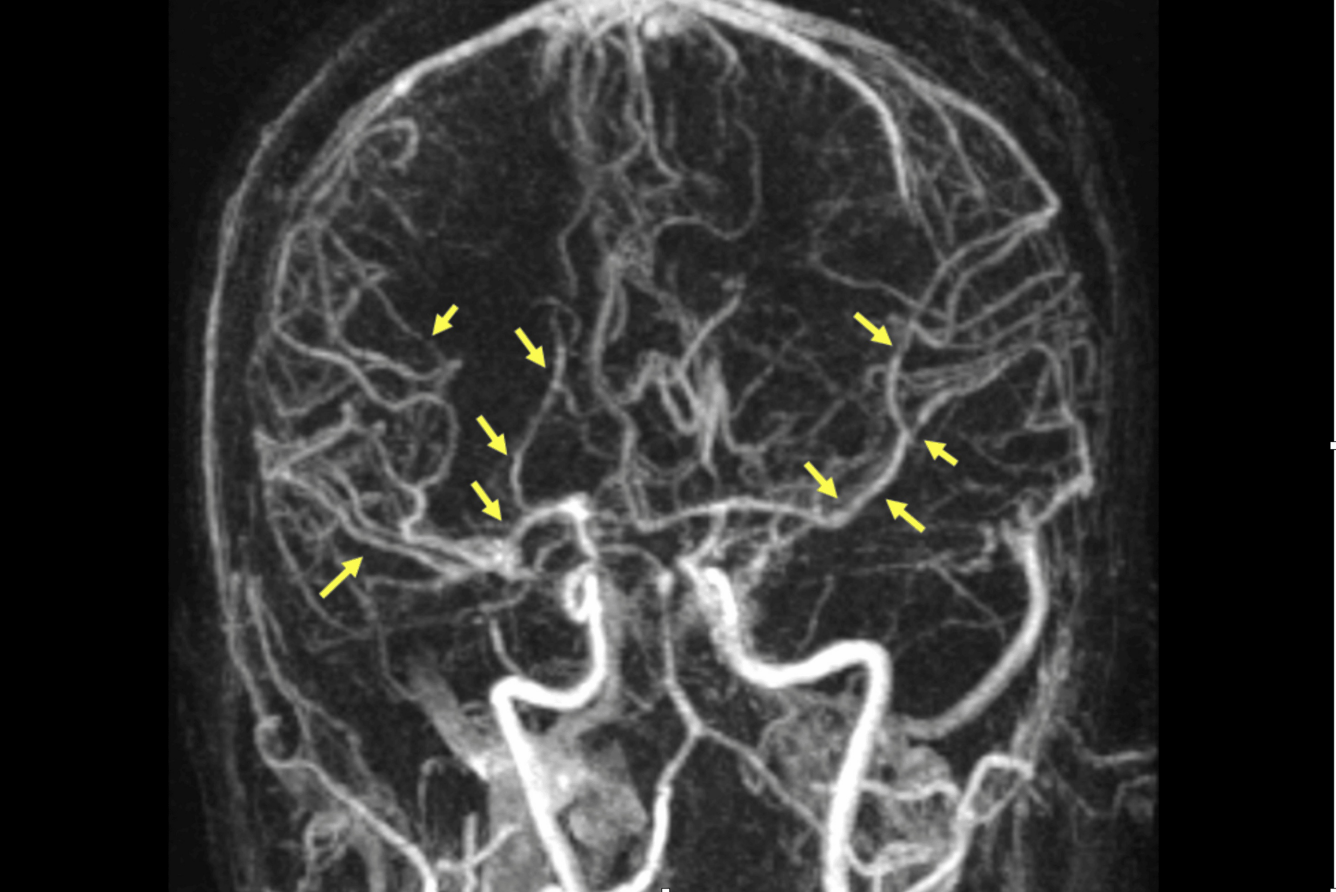
Magnetic resonance angiography of the brain Multifocal-segmental irregularity and narrowing of the anterior circulation are observed bilaterally distal to the A1 segments and middle cerebral artery bifurcations (yellow arrows).

On hospital day three, her course was complicated by acute onset of left hemiparesis and hemispatial neglect, with imaging showing increased prominence of multifocal infarcts. Conventional angiogram and MR angiogram showed multiple diffuse segmental stenoses in the bilateral middle cerebral artery (MCA) and anterior cerebral artery (ACA) territories and severe stenosis of the proximal left subclavian artery and right vertebral origin, prompting rheumatology consult for evaluation of systemic vasculitis (Figure 1). The patient had no history of oral or genital ulcers, jaw claudication, or hearing loss. On exam, temporal artery pulses and pathergy tests were normal. Bilaterally, Bruits were audible over subclavian arteries, with a diminished left radial pulse. Extensive infectious, hypercoagulable, and autoimmune workup was negative (Table 1).

**Figure 2:**
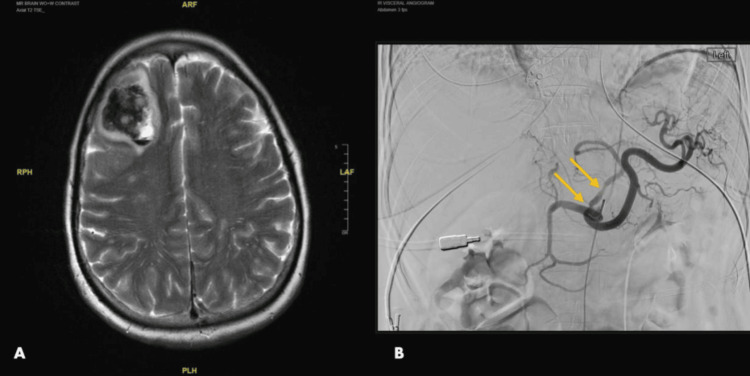
Magnetic resonance angiography of the brain and abdomen (A) Serial imaging on day four showed a new large right frontal hematoma and scattered punctate hemorrhages. (B) A repeat angiography on hospital day seven showed a beaded appearance of the right intrahepatic artery, the left gastric artery (pictured above; solid arrows), multiple pancreaticoduodenal arteries, and the infrarenal aorta, consistent with extracranial involvement.

Methylprednisolone 1g IV daily was started on hospital day four. However, serial imaging on days four and seven showed interval progression intracranially and in intraabdominal vessels (Figure 2). Given her high mortality risk, she was started on plasma exchange (PLEX) and oral cyclophosphamide for possible immune-mediated undifferentiated large- and medium-vessel vasculitis. Notably, there was no further vasculitic progression after the initiation of PLEX.

Brain biopsy of the right dura and frontal lobe showed a perivascular predilection of inflammation, suggesting an angiitic component but no overt vessel wall necrosis. The features were insufficient for a definite diagnosis of CNS vasculitis. Furthermore, CSF protein is not significantly high enough to support CNS vasculitis. Given the extensive, atypical pattern of medium- and large-vessel involvement, clinical suspicion for an infectious precipitant in the setting of dental procedure was high. Blood cultures remained negative. Infectious disease was consulted, and NGS of cell-free DNA from plasma (Karius Test^TM^, Redwood City, CA) was performed, which revealed 72 copies/mL of *Prevotella melaninogenica* DNA (normal: < 10 copies/mL). This supported the possibility of bacteremia leading to immune-mediated vasculitis.

Ceftriaxone was administered for one week, and the patient's course stabilized. She was discharged on day 18 with a slow steroid taper and monthly cyclophosphamide IV. Clinically, the patient had severe motor deficits in her left upper and lower extremities (3/5 strength), left hemispatial neglect, and marked expressive aphasia. However, by the fifth month, she displayed a near-complete resolution of left-sided weakness and was communicating appropriately. She was transitioned to maintenance therapy following six months of cyclophosphamide.

## Discussion

Our case describes a rapidly progressive, infection-induced large- and medium-vessel vasculitic process following a dental procedure that presented atypically for classic rheumatic vasculitides. Many human studies and animal models have suggested that various infectious agents play a role in the development of vasculitis in susceptible individuals. Nevertheless, the relationship between infection and vasculitis is highly complex and remains only partially understood [6].

The processes through which infectious pathogens cause vascular inflammation can be divided into two main categories: direct pathogen invasion of the vessel wall and immune-mediated damage to the vessel wall. Pathogens can directly damage the vessel wall by triggering smooth muscle cell (SMC) proliferation and migration, preventing SMC apoptosis, causing endothelial dysfunction (promoting procoagulants and inhibiting vessel dilation), and increasing the production of reactive oxygen species, cytokines, chemokines, and cellular adhesion molecules [7]. Immune-mediated vascular damage induced by microorganisms is proposed to occur through a complex interplay involving immune complexes, molecular mimicry, cytokine secretion, superantigens, and T-cell-mediated injury [7].

This case illustrates a rapidly progressive, infection-triggered large- and medium-vessel vasculitis following a dental procedure. The patient's presentation was atypical for classic rheumatologic vasculitides such as giant cell arteritis (GCA), Takayasu arteritis (TAK), or polyarteritis nodosa (PAN), for reasons detailed below.

GCA is a systemic inflammation targeting medium and large vessels, particularly the extracranial branches of the carotid arteries [8]. This disease is two to three times more frequent in women compared to men and predominantly affects those over 50 years old [8]. Typical symptoms of GCA include headaches, tenderness in the temporal artery, acute vision loss in 5-15% of patients, strokes in 3-7%, jaw pain during chewing, polymyalgia rheumatica (PMR), and low-grade fever [8]. The patient in our case did not meet the criteria for GCA as defined by the 1990 American College of Rheumatology criteria or the 2022 EULAR (European Alliance of Rheumatic Diseases) recommendations [9]. Her neurological deficits were too acute without prodromal symptoms, she lacked associated PMR, and her vascular involvement included the atypical posterior cerebral artery, which is rarely affected in GCA.

TAK is an uncommon autoimmune vasculitis that affects the aorta and its major branches, as well as the subclavian and carotid arteries [10]. Fever was the most frequent symptom, followed by chest pain, claudication, headache, shortness of breath, weight loss, syncope, night sweats, and unequal pulses and BP [11]. TAK was also unlikely, given the acute onset and lack of upper extremity claudication or vascular bruits.

PAN is a type of necrotizing vasculitis that mainly targets medium-sized blood vessels and can also affect smaller vessels [12]. General, non-specific symptoms such as asthenia, fever, weight loss, myalgia, and arthralgia are frequently the initial symptoms of PAN (85-93%) [12]. Neurologic manifestations occur in more than two-thirds of patients, most commonly as motor and sensory mononeuritis multiplex of the peripheral nerves (59-79%) [13]. PAN was initially considered due to the medium-vessel involvement seen on imaging in our case. However, her initial stroke-like presentation and lack of weight loss, abdominal pain, or peripheral neuropathy made PAN criteria tenuous. The rapidity of her deterioration and the diffuse, multi-territory large- and medium-vessel infarcts were highly atypical for PAN.

Given the overall atypical presentation not conforming to known rheumatologic entities and the clear nidus following her recent dental infection, an infectious etiology was strongly suspected despite negative cultures. NGS of cell-free DNA identified *Prevotella melaninogenica*, an anaerobic oral commensal. Many *Prevotella* species can contribute to oral inflammatory processes [14]. While typically causing local dental/periodontal disease, *Prevotella* species have been rarely implicated in many pathogenic conditions inside and outside the mouth, precipitating systemic vasculitis [14,15].

Given our patient's successful response to PLEX and immunosuppression, we suspected an immune-mediated mechanism rather than a direct pathogen invasion. However, traditional culture techniques failed to identify an infectious trigger. The emergence of NGS technologies has enhanced diagnostic capabilities for culture-negative infections [16]. The Karius Test^TM^ is a commercially available assay that sequences cell-free DNA from plasma to identify pathogen-derived sequences. Applying this assay revealed *Prevotella melaninogenica* DNA, an anaerobic gram-negative oral commensal implicated in periodontal and dental infections. While the detection of low-level bacteremia raises concerns for clinical insignificance, the patient's severe systemic vasculitis developing shortly after a dental procedure pointed to this finding being pathogenic.

Our case adds to the limited reports of *Prevotella* species causing vasculitis and highlights how NGS diagnostics can elucidate atypical clinical presentations by detecting fastidious pathogens. As NGS becomes more widespread, our understanding of infection's role in vasculitis pathogenesis will grow. *Prevotella* is a Gram-negative anaerobic bacilli, and positivity for *P. melaninogenica* was high in chronic periodontitis compared to healthy [17].

The patient's dire condition necessitated empiric treatment with PLEX, although evidence for its use in vasculitis is limited. Cyclophosphamide was chosen for rapid immunosuppression over rituximab, as her condition was too fulminant to await B-cell depletion [18]. Methotrexate was later added as a steroid-sparing agent. Her remarkable recovery, initially thought improbable, illustrates the importance of an open diagnostic approach when presentations defy established disease patterns.

## Conclusions

We conclude that *Prevotella* bacteremia following dental procedure, without endocarditis, was the probable cause of this patient's vasculitis. The case highlights the potential benefit of NGS tests such as Karius in clinical settings for atypical cases with high suspicion for infectious etiology. It also demonstrates the challenging diagnosis and management of undifferentiated vasculitides, which may require multidisciplinary input from rheumatology, neurology, and infectious disease. Ultimately, identifying and treating an underlying trigger is crucial for improving outcomes in these complex cases.
